# Dentin-like tissue formation and biomineralization by multicellular human pulp cell spheres *in vitro*

**DOI:** 10.1186/1746-160X-10-25

**Published:** 2014-06-20

**Authors:** Jörg Neunzehn, Marie-Theres Weber, Gretel Wittenburg, Günter Lauer, Christian Hannig, Hans-Peter Wiesmann

**Affiliations:** 1 Technische Universität Dresden, Institute of Material Science, Chair for Biomaterials, Budapester Strasse 27, D-01069 Dresden, Germany; 2 Department of Restorative and Pediatric Dentistry, University Hospital Carl Gustav Carus, Fetscherstrasse 74, D-01307 Dresden, Germany; 3 Department of Oral and Maxillofacial Surgery, University Hospital Carl Gustav Carus, Fetscherstrasse 74, D-01307 Dresden, Germany

**Keywords:** Biomineralization, Dental pulp cells, Tissue formation, Pulp spheres, Pulp tissue regeneration

## Abstract

**Introduction:**

Maintaining or regenerating a vital pulp is a preferable goal in current endodontic research. In this study, human dental pulp cell aggregates (spheres) were applied onto bovine and human root canal models to evaluate their potential use as pre-differentiated tissue units for dental pulp tissue regeneration.

**Methods:**

Human dental pulp cells (DPC) were derived from wisdom teeth, cultivated into three-dimensional cell spheres and seeded onto bovine and into human root canals. Sphere formation, tissue-like and mineralization properties as well as growth behavior of cells on dentin structure were evaluated by light microscopy (LM), confocal laser scanning microscopy (CLSM), scanning electron microscopy (SEM) and energy dispersive X-ray spectroscopy (EDX).

**Results:**

Spheres and outgrown cells showed tissue-like properties, the ability to merge with other cell spheres and extra cellular matrix formation; CLSM investigation revealed a dense network of actin and focal adhesion contacts (FAC) inside the spheres and a pronounced actin structure of cells outgrown from the spheres. A dentin-structure-orientated migration of the cells was shown by SEM investigation. Besides the direct extension of the cells into dentinal tubules, the coverage of the tubular walls with cell matrix was detected. Moreover, an emulation of dentin-like structures with tubuli-like and biomineral formation was detected by SEM- and EDX-investigation.

**Conclusions:**

The results of the present study show tissue-like behavior, the replication of tubular structures and the mineralization of human dental pulp spheres when colonized on root dentin. The application of cells in form of pulp spheres on root dentin reveals their beneficial potential for dental tissue regeneration.

## Introduction

The root canal treatment is a way of preserving a tooth by removing contaminated or injured dental tissue, disinfecting the canal system as well as obturating and sealing it with synthetic material. Over the past decade, the success rate of endodontic treatments was greatly increased due to advancements in dental materials, antimicrobial therapy and endodontic technology [1,2].

However, endodontically treated teeth are lacking blood and nervous system supply. This results in the loss of sensing environmental changes such as caries progression and distinction of temperature differences [2]. Furthermore, those teeth are more vulnerable to masticatory forces since the ability to perceive tactile stimuli is diminished due to a reduction of mechanoreceptors when removing the pulp tissue [3]. The lack of pulp tissue and the containing odontoblasts inhibit reparative dentin formation, which is particularly important for the protective self-defence-system of the tooth [4].

Besides the endodontic approach, injuries of the coronal pulp caused by trauma and deep carious lesions resulting in pulp exposure still mark a major challenge. In consideration of these concerns, maintaining or regenerating pulp vitality is a preferable goal in current endodontic research.

The revascularization of the tooth during root maturation has been investigated by several studies focussing on trauma patients [5,6]. One promising approach is cell therapy with pulp stem/progenitor cells directed by morphogens [7]. Thereby, a pronounced regenerative potential of the pulp-dentin-complex was observed, which was ascribed to the multipotency of pulp stem cells [8]. Based on their embryologic origin pulp fibroblasts and stem cells are able to differentiate into ecto- and mesodermal cell types causing them to be of special interest in tissue engineering. The human dental pulp contains cells such as stem cells, fibroblasts, undifferentiated mesenchymal cells, odontoblasts and defence cells such as histiocytes, macrophages, granulocytes, mast cells and plasma cells. Additionally, an extensive vascular supply and the nerv plexus of Rashkow are located within the pulp chamber that have important functions in inflammatory events and subsequent tissue repair.

In vivo studies revealed the possible use of dental pulp stem cells in the regeneration of various tissues. Further studies confirm that the differentiation of stem cells as well as angiogenesis and neurogenesis are essential for pulp regeneration [9].

Therefore, scaffolds, cells and bioactive molecules are essentially needed for dental tissue engineering. A wide variety of scaffolds such as collagen, fibrin, synthetic matrices or other hydrogels are frequently used for this purpose [10,11]. In this context, dentin specimens are suitable for *in vitro* experiments to evaluate the efficacy of these strategies. Pulp cells seeded onto pre-treated dentin surfaces had a proliferation rate similar to that of pulp cells on two-dimensional controls; in addition, they exhibited multipolar processes extending into dentinal tubules [12,13]. Another study showed the same extension of DPC processes into dentinal tubules, which proved their odontoblastic phenotype after being inoculated onto dentin discs [14]. The studies mentioned above indicate that not only the composition of dentin but also its topography, in this case dentinal tubules, might play a key role in cellular differentiation of the DPC [15].

There are contrasting results regarding the seeding efficiency of DPC on scaffolds. While suspension cells are routinely used for dental cell biology in two-dimensional systems, it is known that micromass cultures have several advantages over suspension cells for tissue engineering approaches. Three-dimensional and tissue equivalent cell agglomerates, so called spheres, show similar cell proliferation and differentiation as tissues *in vivo*. In different studies micro mass cultures of osteoblasts, osteoblast-like cells and other cell types such as stem cells [16,17] were investigated intensively and affirmed the tissue equivalent cell behavior [18].

3-D cell-culture-systems and especially the use of micromass cultures have proven themselves as a potent method when being used as *in vitro* testing systems due to their tissue-like behavior. Furthermore, these cell-culture-systems were also employed for biomaterial testing and could probably be used directly as an already pre-differentiated tissue unit for tissue regeneration [18-21].

The use of pulp spheres containing DPC could have an advantage over previous restoration methods, where cells had to be connected to a scaffold in order to be placed into a prepared root canal for pulp-tissue engineering. Using pulp spheres, however, it is possible to insert DPC scaffold-free into root canals. Furthermore, the three-dimensional cultivation method of the spheres enables a pre-differentiation of the DPC into different kinds of tissue for a faster formation of pulp tissue before the cells are placed into the root canal. An application of these pre-differentiated pulp spheres into prepared roots for tissue engineering, but also during a partial pulp removal is conceivable.

Therefore, spheres containing DPC derived from human wisdom teeth were applied onto bovine root dentin and into human root canals as an *in vitro* test system for the first time. The aim of this study was to investigate the aptitude of these micromass cultures, spheres, regarding tissue engineering and pulp regeneration on root dentin and in root canals from a morphological and structural perspective.

## Materials and methods

### Bovine and human root canal model preparation

**Figure 1:**
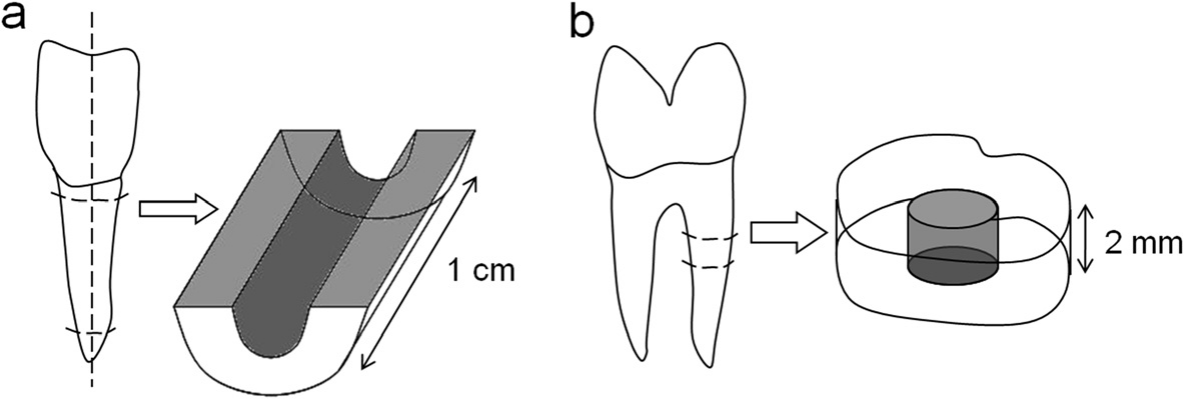
**Preparation of bovine and human root canal models.** Bovine roots were sectioned longitudinally and the root canal surface was treated with a rose-bur **(a)**. Additionally, human roots were cut horizontally into 2 mm thick discs after root canal preparation **(b)**. Both models underwent ultrasonic desorption to remove the accumulated „smear layer“(abrasive dust, debris).

To investigate the behavior of human dental pulp spheres on dentin *in vitro* two different root canal models were established*.*A bovine root canal model was prepared to reveal the spheres behavior on a vast root dentin surface (Figure 1a). Therefore, the pulp tissue of extracted bovine incisor roots of two-year-old cattle (Südost Fleisch GmbH, Altenburg, Germany) was extirpated and the roots were divided in half, longitudinally with an average canal length of about 1 cm, using a precision table top cut-off machine (Accutom-50, Struers). Additionally, the root canals were treated with a rose-bur (low-speed: 1000 rpm) to remove residual pulp tissue, denticles as well as sclerotic zones in the root dentin and to simulate a root canal preparation with files.A human root dentin canal model served to examine the sphere behavior on a more tight and physiological bases (Figure 1b). Incisors, premolars and molars of middle-aged donors (in the age of 25–50) were extracted during routine surgical treatment and underwent a root canal preparation using ProTaper (Dentsply Maillefer, Ballaigues, Switzerland) and sodium chloride 0,9% (NaCl) as irrigant. The roots were explored and prepared up to ProTaper finishing file F5 with a 0.50 mm diameter and a fixed taper of 5% in its apical extent. Afterwards, the endodontically treated roots were cut horizontally into 3 mm thick discs. The accumulated „smear layer“(abrasive dust, debris) in the bovine as well as the human root canals was removed by ultrasonic desorption using 70% EtOH, 3% EDTA and distilled water for one minute each, respectively. Subsequently, the bovine root canal specimens were sterilised to prevent a possible contamination of the spheres.

### Cultivation of human dental pulp cells

Pulp tissue derived from human wisdom teeth was macerated enzymatically with collagenase in order to isolate the cells from the surrounding tissue. According to facs analysis approximately 80% of the isolated cells showed stem cell character. The DPC were cultivated up to the fourth passage in D-MEM (low glucose), 20% FCS, 2% HEPES, 100 U/ml penicillin, 100 μg/ml streptomycin, 50 μg/ml gentamicin, 2,5 μg/ml amphotericin B (all PAA, Cölbe, Germany) at 37°C, 5% CO_2_ and 95% humid atmosphere with a medium change two to three times per week. The used cells were tested positive for their potential to be differentiated in an osteogenetic and angiogenetic way as described in literature [22].

### Harvesting of human dental pulp cells and sphere formation

DPC were harvested by trypsin incubation, counted using CASY cell counting technology (Schärfe System GmbH, Reutlingen, Germany) and transferred to a non-attachment environment. For this purpose, chambers of 96-well plates were prepared by applying 50 μl of a mixture of 20 mg/ml agarose (Biozym Scientific GmbH) in DMEM (Biochrom)/HGEM per well. A population of 100 000 cells per well was seeded into the treated cell culture dishes and incubated in the medium mentioned above at 37°C and 8% CO_2_. The medium was changed twice per week. In preliminary tests it was possible to differentiate the cultivated human pulp cell spheres in different tissue specific ways to validate their multipotent stem cell character as described previously [23].

### Cultivating pulp spheres on biological environment

In order to evaluate the potential of the pulp spheres to interact with a physiological environment, five-day-old pulp cell spheres were aspirated with a sterile pipette and transferred to be cultured on ten prepared halved bovine roots and into eleven human root canals, respectively.

Depending on the root canal size, two or three spheres were seeded onto bovine dentin. They were located far enough from each other, so that the cells that started to grow out of the one sphere would not disturb the cells which grew out of the other spheres (with a distance of 3 mm between each other). In case of the human root canals, two spheres were seeded into one canal to ensure a contact between the spheres inside the canal to evaluate the sphere interaction in a narrow and physiological environment.

The sphere-seeded material was evaluated during the whole trial period by light microscopy (LM) and after seven days and 28 days by scanning electron microscopy (SEM), respectively. As a reference and for LM- and confocal laser scanning microscopical-analytics (CLSM), five cell spheres were cultivated on polystyrene culture dishes and were also evaluated after seven days. The cell-seeded specimens and culture dishes were cultivated as described above.

### Light microscopy

The pulp spheres were investigated by light microscopic analysis (LM) during the whole trial period by the use of a Zeiss Axiovert 40 CFL combined with a Canon PowerShot G11. This method was mainly used during the examination of spheres grown in root canal models to monitor potential contamination and to notice unphysiological cell development.

### Scanning electron microscopy

For scanning electron microscopic investigation (SEM), pulp spheres were cultivated on the dentin and fixed with glutaraldehyde, followed by dehydration in an ascending series of isopropanol, and chemical drying through the iterative transfer into hexamethyldisilazane (HMDS). The samples were fixed on SEM stubs and sputtered with gold-palladium. Scanning electron microscopy was carried out using a Philips ESEM XL 30 in Hi-Vacuum mode by detecting secondary electrons for imaging and by detecting backscattered electrons to investigate material contrasts. For a more detailed material investigation an energy dispersive X-ray spectroscopy (EDX) was performed.

### Confocal laser scanning microscopy

To evaluate sphere formation, cell-cell-contacts inside the cell agglomeration and cell performance of the outgrown cells, five-day-old pulp spheres were stained with actin, focal adhesion contacts (FAC) and 4′,6-diamidino-2-phenylindole (DAPI, Sigma) for confocal laser scanning microscopy (CLSM).

After washing and fixing, the cells were permeabilized with 0.2% Triton-X-100 in PBS and blocked with 1% bovine serum albumin (BSA, Sigma) for 30 min. FAC were stained with AlexaFluor 488-Phalloidin (Invitrogen), cytoskeletal actin was stained with AlexaFluor 546 and cell nuclei were stained with DAPI.

Microscopy was carried out by the use of an upright Axioscop 2 FS mot equipped with a LSM 510 META module (Zeiss, Jena, Germany) controlling an argon-ion (Ar+) laser, helium-neon (HeNe) laser and NIR-femtosecond titanium-sapphire laser for 2-photon excitation (Coherent Mira 900 F). The excitation of AlexaFluor 488 was carried out at 488 nm (Ar + laser) and the excitation of AlexaFluor 546 at 546 nm (HeNe laser). The NIR-fs-laser was used for the excitation of DAPI at 750 nm (2 photon excitation) and fluorescence was recorded at 461 nm.

## Results

**Figure 2:**
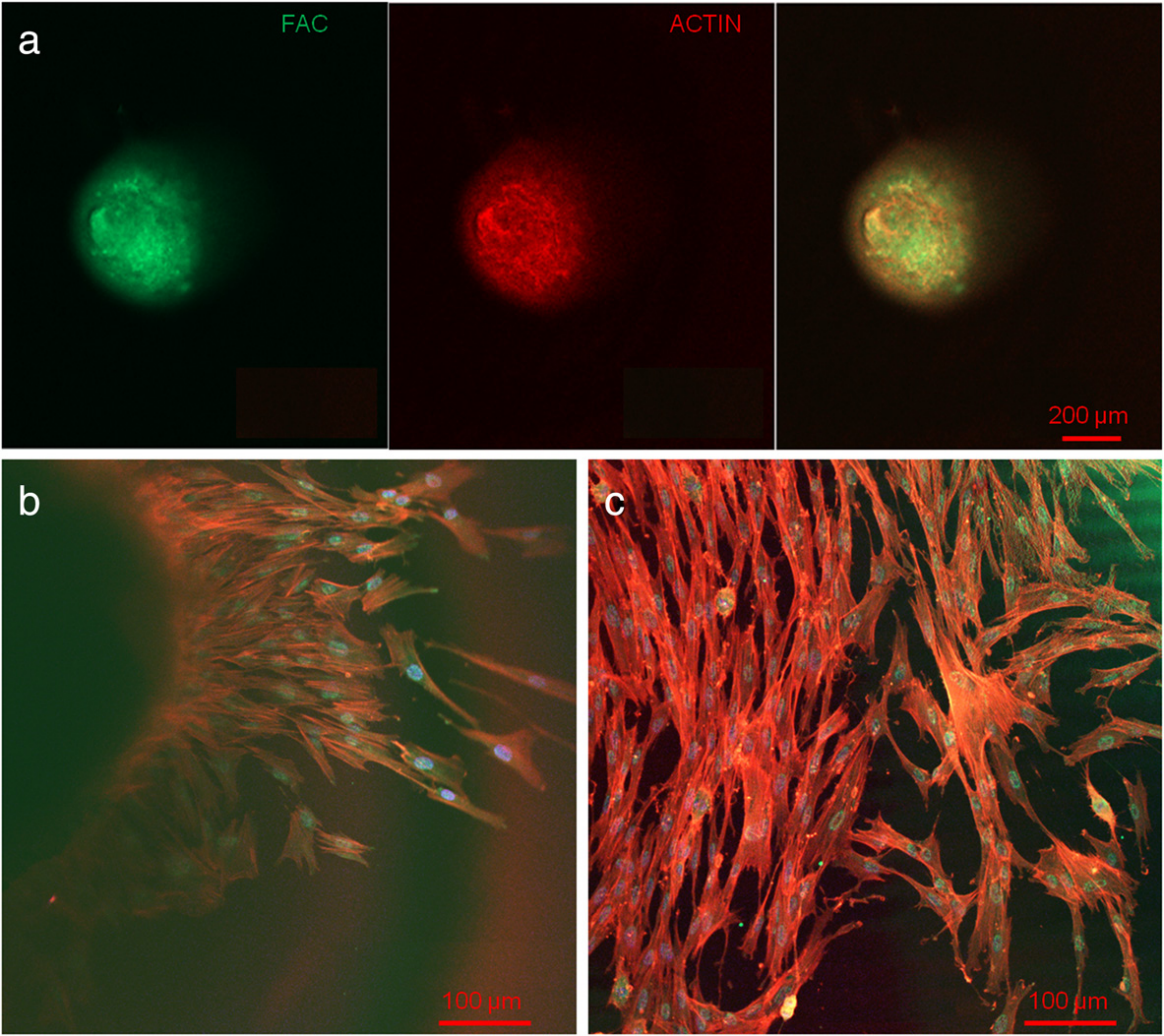
**Confocal laser scanning microscopic evaluation.** Actin (red), FAC (green) and DAPI (blue) stained cells in the upper third of a pulp sphere **(a)** and of its outgrown cells **(b** and **c)** representing a dense actin formation and consistent round cell nuclei.

**Figure 3:**
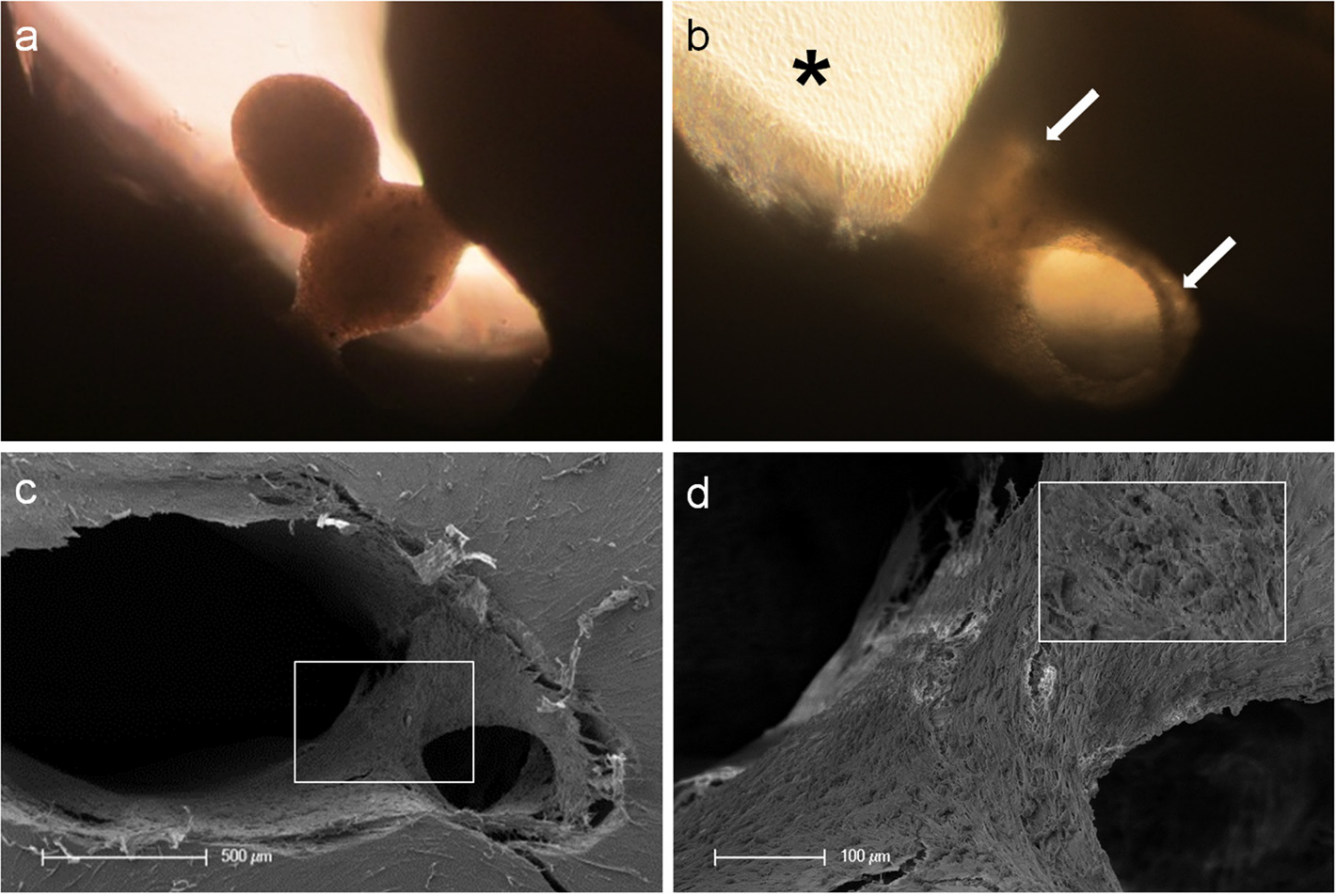
**Tissue-like behavior of human pulp sphere cells.** Figure **(a)** shows merging pulp-spheres in a human root canal model after five days. After 28 days of cultivation the two spheres merged, cells grew out of the spheres and coated the root dentin walls (white arrows). **(b)** Outgrowing cells also covered the underlying polystyrene culture dish and built up a dense cell layer (black asterisk). SEM investigation of the merged spheres **(c** and **d)** represents the even surface of the new cell construct and the widespread coverage of the root canal walls **(c)** as well as the randomly distributed different sized particles **(d)**.

Light microscopic evaluation during sphere cultivation showed cell and sphere development without any contamination and a fast and complete formation of round cell spheres after five days.After sphere formation, confocal laser scanning and light microscopic investigations illustrate a round shape (Figure 2a) with evenly plain surface (Figure 3a). The results of the actin and FAC staining, as well as CLSM-investigations showed a very high expression of these factors inside the spheres. Both actin and FAC were equally allocated in the spherical cell construct building up a dense network (Figure 2a).

**Figure 4:**
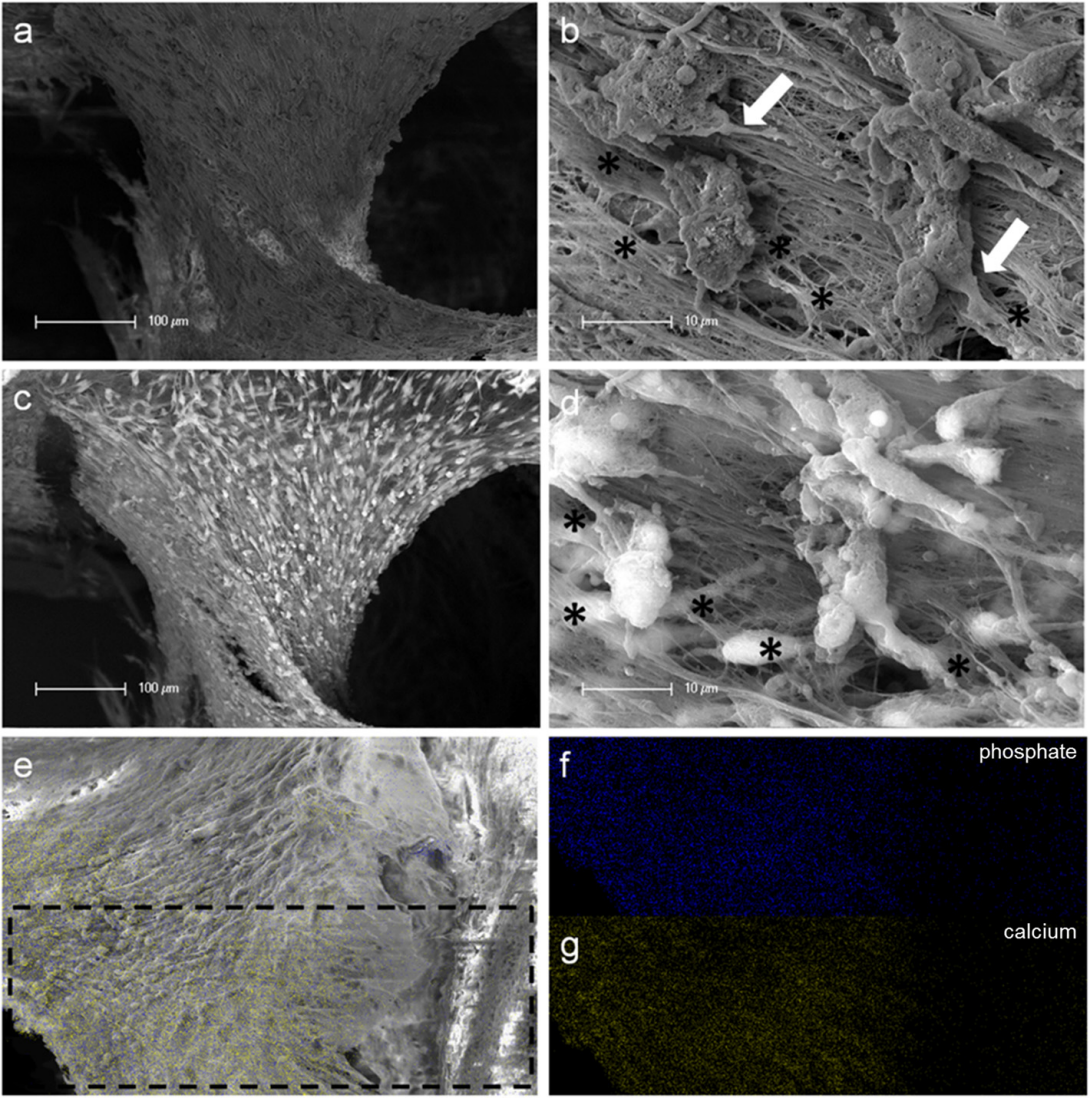
**SEM and EDX investigation of merged human pulp cell spheres in a human root canal.** Figure **(a)** shows a fibrillar surface of the closed cell construct with different sized mineral like structures on it (Figure 4b) by detection of secondary electrons. The white arrows in figure **(b)** represent a cellular connection between the mineral particles and the underlying cell layer. The images in figure **(c)** and **(d)** show the same areas investigated by the detection of back scattered electrons with a clearly seen atomic number depending material contrast between the spheres’ cell matrix and the mineral particles. Black asterisks in figure **(b)** and **(d)** denote particles with higher atomic numbers, which could only be seen in figure **(d)** by the aid of the material contrast. The area of the sphere, which was investigated by EDX, is marked with black lines in figure **(e)**. The results concerning the higher concentration of mineral specific elements are represented in figure **(f)** (phosphate) and figure **(g)** (calcium).

**Figure 5:**
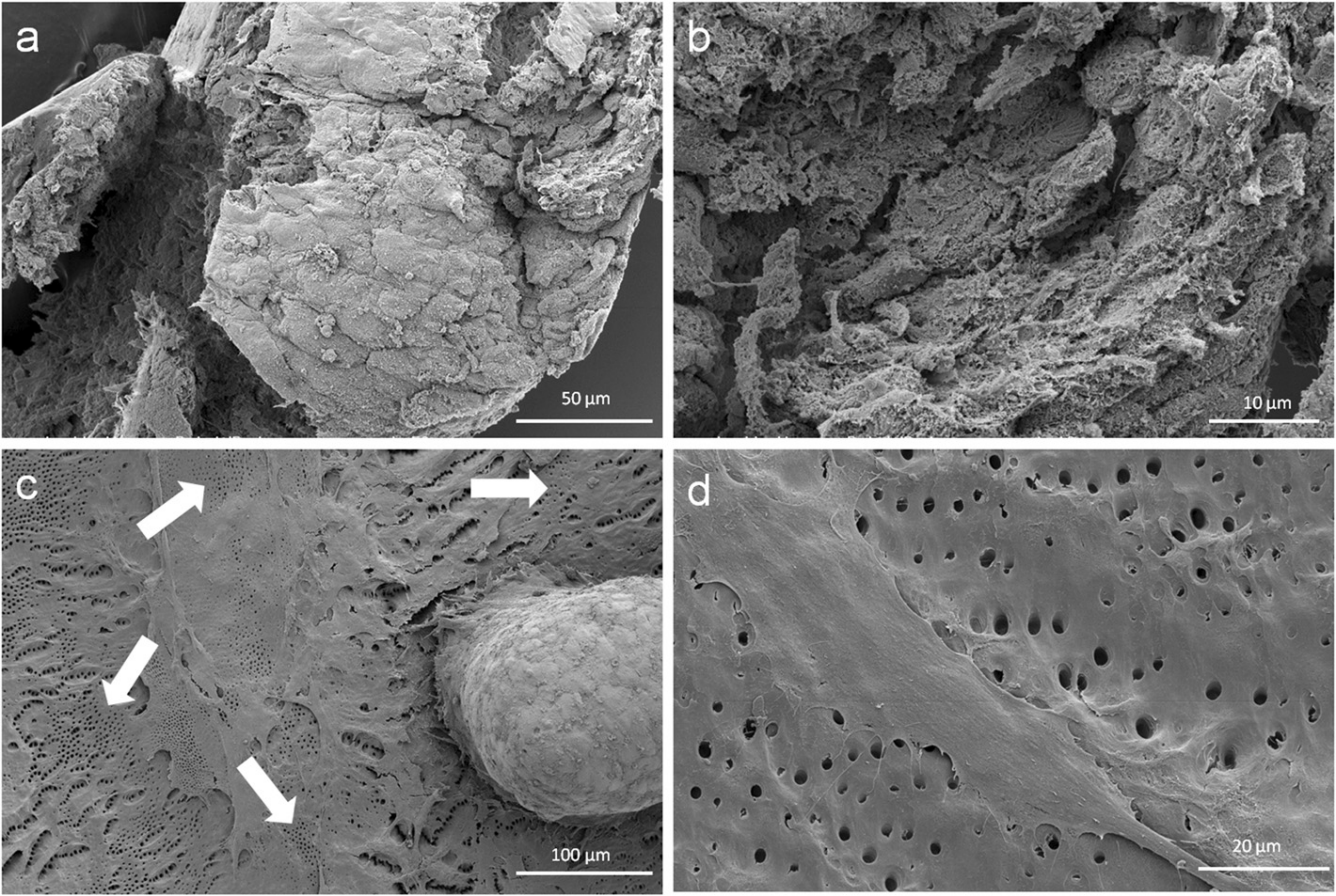
**Scanning electron microscopic sphere and cell investigation.** SEM images of the outer epithelial-like shell of a cell sphere **(a)** and the less organized cells with a higher production of extracellular matrix inside the sphere **(b)**. Figure **(c)** shows a pulp sphere attached onto bovine dentin with a high number of outgrowing, multilayered cells as well as flat spread out and attached cells on bovine root dentin **(d)**. The white arrows in figure (4c) highlight the dentin tubule-like holes in the cell multi-layer.

**Figure 6:**
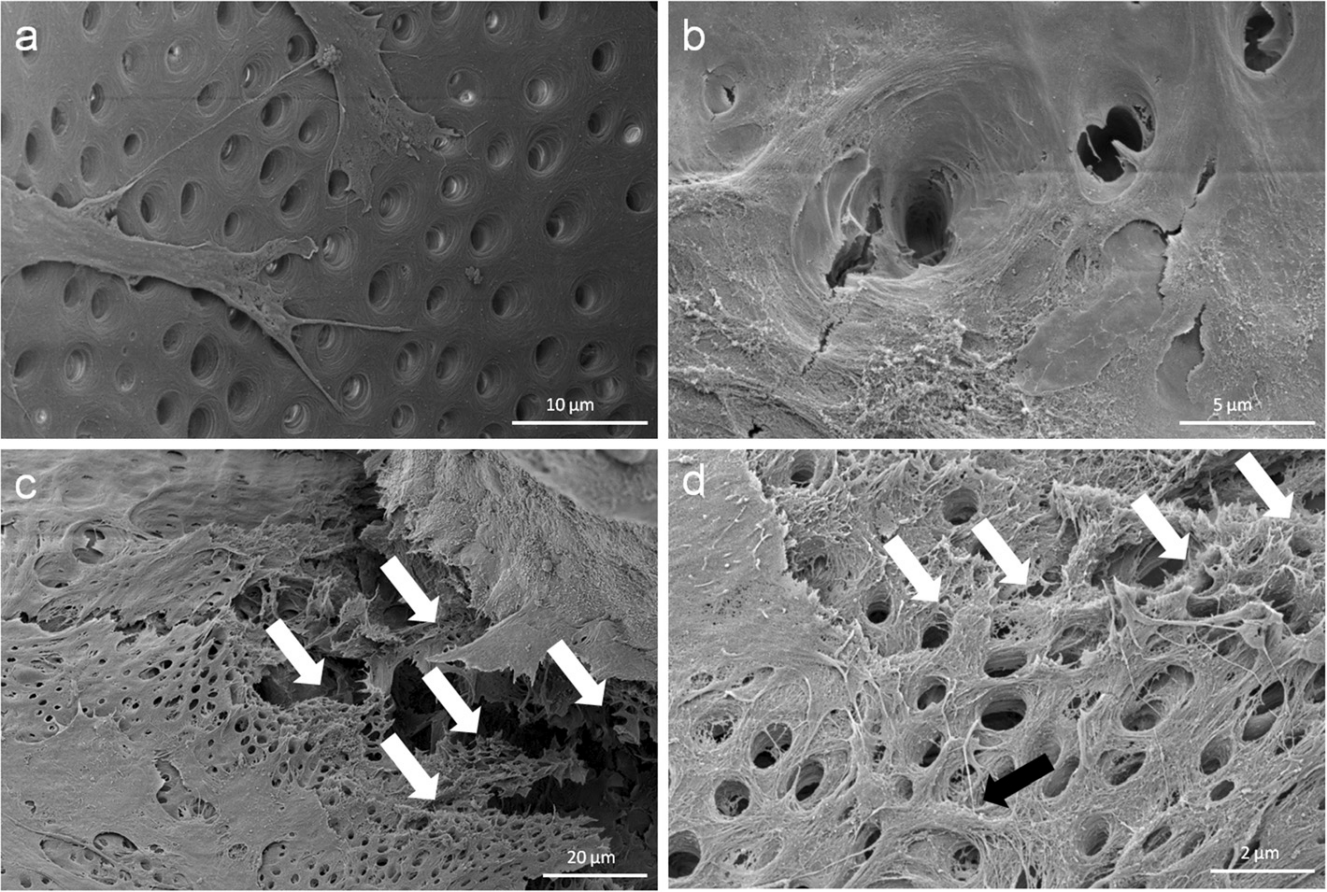
**Cell behavior on bovine dentin.** Scanning electron microscopic images show the bovine dentin surface orientated migration of outgrown pulp sphere cells **(a)** and the vortical ingrowth into the dentinal tubules as well as the covering of the tubular walls by these cells **(b)**. Multilayered cell formations with cellular emulation of three-dimensional dentin structures could be detected in the multi layered outgrowth area adjacent to a sphere (white arrows in Figure 6**c**). The white arrows in figure **(d)** denote the fibrillar dentin tissue-like structure over numerous cell layers (black arrow).

At the contact area between the pulp sphere and an untreated cell culture dish surface, the cells grew out of the sphere as described in literature [18]. The outgrown cells migrated on the polystyrene substrate and presented a pronounced actin skeleton (red) with FAC (green) and consistent round cell nuclei dyed blue (Figure 2b and c).Nearby lying pulp spheres in the human root canals aligned themselves towards each other and merged into bigger tissue-like cell formations containing multiple cell layers (Figure 3a). Figure 3a represents the intense contact between two adjacent spheres and the surrounding human root dentin.After 28 days, the two fused spheres expanded to a homogenous tissue unit. The light microscopic evaluation indicated a nearly complete union of the spheres in the lumen of a human root canal (Figure 3b). The newly built cell tissue branched out and covered the human root canal walls (Figure 3b white arrows, Figure 3c). Other cells, outgrown from the spheres, attached on the polystyrene cell dish surface, migrated and formed a dense cell layer (Figure 3b *).SEM images in Figure 3c and d represent the homogeneous surface of the two merged spheres with numerous different sized particles (zooming in Figure 3d).The SEM investigation showed a fibrillar surface of the closed cell construct of the merged spheres (Figure 4a) with different sized mineral like structures on it (Figure 4b). These mineral particles seem to be connected with the cell matrix (white arrows in Figure 4b). The back scattered electrons (BSE) represented a very clear, atomic number depending material contrast between the spheres cell matrix and the mineral particles (Figure 4c and d). Especially Figure 4c represents the very large number of biomineralized particles.A further result of the SEM investigation illustrates the different localization of the mineral particles. The black asterisks in Figure 4b and d point out the detections of the particles with a higher atomic number as the cell material, which is not visible in the secondary electron image with a topographical contrast of the same area (Figure 4b). In this image these particles are covered with cell matrix. This effect is also seen in Figure 4c. The shining particles are represented in two different ways. In some areas the edges seem to be definite from the surrounding cell material (middle part of Figure 4c). In other regions they appear to look pale and blurred (in the upper part of Figure 4c).The additionally performed mineral proof by EDX (Figure 4e-g) revealed a high concentration of phosphate (Figure 4f) and calcium (Figure 4g) on the newly build cell agglomerate.SEM images of a mechanically broken pulp sphere after seven days of micromass formation revealed the integrity of highly aggregated pulp cells inside the sphere (Figure 5a,b). The smooth surface of the spheres seemed to be constituted by epithelial-like cell layers that are superimposed by each other with distinctive cell-cell contacts (Figure 5a). Cells in the inner region of the spheres appeared to be less organized. These cells seemed to interact with each other in clearly visible cell-cell-contacts and with a high formation rate of extracellular matrix (Figure 5b).The pulp spheres seeded onto bovine dentin showed a very intense contact with the biological substrate. Figure 5c and d represent the widespread contact area between the spheres and the dentin surface. In this specific contact area, a high number of cells grew out of the spheres in multiple layers (Figure 5c). The whole area, surrounding the spheres was covered by multilayered cells. The majority of this area was covered with small, tubule like vents (Figure 5c “white arrows”). On the dentin, the migrated cells aligned themselves on the physiological surface, representing a very intense cell adhesion, which is indicated by the flat spread out cell bodies on the dentin (Figure 5d).Furthermore, pulp cells partially extended into dentinal tubules immediately after growing out of the cell spheres (Figure 6a). After 28 days, a number of dentinal tubular walls were covered by ingrown pulp cells. The cells grew vortically into the porous structure (Figure 6b).Especially, in the areas directly adjacent to the spheres, the multilayered outgrown cells emulated dentin structures with newly constructed tubule like formations (Figure 6c). The white arrows in Figure 6c and d indicate the formation of this dentin like structure over several cell layers in a preparation conditioned split between cell layer and sphere. Figure 6d also represents the fibrillar constitution of this structure built up by cells (black arrow).

## Discussion

Within the last years, cells derived from pulp-tissue were investigated concerning their multipotent stem cell character as well as their ability to differentiate in diverse ways such as angiogenetic or osteogenic differentiation [24]. In this study, the possibility of human dental pulp cells (DPC) serving as potential progenitors for odontoblast formation and biomineralization initiators was investigated. Several studies displayed the high potential of DPC regarding this matter [22,24-26]. Besides the possibility to influence the specific differentiation of these cells, an odontoblast-like phenotype, structure, cell formation and differentiation behavior were shown in *in vitro* and *in vivo* setups [25-27].

The results of the present study revealed the same differentiation characteristics of the adapted cells on the dentin models as postulated in literature mentioned above. The DPC used in this study proved a stem cell-like character if differentiated angiogenically and osteogenically. Furthermore, a cultivation of the cells over various passages without losing the differentiating potential was possible.

During the last years, the aim in several studies was to use cells derived from pulp tissue to induce a regeneration of the pulp. Hitherto, the cells have been seeded on different scaffold materials such as organic collagen, chitosan, hydroxyapatite/tricalcium phosphate (HA/TCP) or inorganic polymer polylactic-co-glycolic-acid (PGLA) in order to support the organization and vascularization of the newly formed tissue [10,28-30]. However, the unpredictable degradation of inorganic as well as organic scaffold materials represents a risk factor concerning wound healing and complete tissue regeneration *in vivo*.

Three-dimensional micromass cultures, so called spheres, are known to construct tissue-like formations and are often used as *in vitro* cell culture systems to test biomaterials and surfaces for their tissue tolerance. These solid, three-dimensional constructs are also described as a potential method for scaffold free tissue engineering [18-21,31]. According to the sole use of DPC-spheres and the absence of additional scaffold materials, negative or non-physiological influences can be excluded almost completely.

In this study, pulp spheres were generated from cultivated cells derived from human pulp tissue. The applicability of pulp spheres for pulp tissue reconstruction was investigated for the first time.

In order to simulate the structure and geometry of the physiological requirements found *in vivo,* sectioned bovine roots and horizontally cut human root canal discs were used as root canal models. After preparation, the surface of the treated root canal models represented evenly distributed dentinal tubules. The smear layer resulting during the preparation was removed via ultrasonic treatment, so that a potential cell growth into the open tubules was possible. The cultivated pulp spheres were distributed homogenously with a distance of approximately 3 mm from each other onto the bovine root canal dentin. Due to the small lumina of human root canals, the spheres were placed within the human dentin discs in a more physiological and tighter condition.

In this study, primarily bovine teeth were chosen for the preparation of the root canal models because of their good availability, processability and comparability. The greater size of bovine roots provides a larger area for experimental procedures [32]. Bovine teeth from animals in the same age category with common genetic ancestry and diet show a more homogeneous mineral composition of the dental hard tissue when compared with teeth gained from humans of different ages and diverse diets. The hardness (KHN = Knoop Hardness Numbers) of human and bovine dentin is quite similar [33]. Also the size and the amount of dentinal tubules per mm^2^ in crown dentin are identical for human molars and bovine incisors [34,35]. However, the diameter of dentinal tubules in bovine root dentin is larger than the diameter of dentinal tubules in human root dentin [34-36]. As mentioned before, the typography of surfaces influences the cell adherence, growth and even the differentiation behavior of the cells. Thus the difference in tubule diameter comparing the decreasing sizes of bovine root dentin tubules and coronal, middle and apical human root dentin tubules was of interest to examine the behavior of the DPC on diverse topographic dentin surfaces. The larger diameter of bovine dentinal tubules could have facilitated the extension of the growing cells into the tubules as compared with human root dentin. Furthermore, the size of the tubule-diameter is also dependent on dentinal sclerosis. Dentinal sclerosis which implies a decrease in size up to a complete blocking of dentinal tubules of root dentin is, unlike dentinal sclerosis in crown dentin, age-related [37,38].

In this investigation, root dentin of two-year-old cattle was used for the root canal models. Hence, the results of this study can only be compared to young human teeth of patients in their mid-twenties when dentinal sclerosis of the root dentin has not yet taken place [38].

In order to observe the behavior of cell spheres in a narrow and physiological *in vivo* like environment in comparison to a flat and larger area provided by the bovine root dentin models, the spheres were also applied into human root canal models. After two days, the spheres already merged and after ten days outgrown cells covered the human dentinal walls. The structure, form and cell orientation of the cultivated spheres, as well as the outer sphere shell and the development of intimate cell-cell contacts and matrix formation of the cells inside the spheres shown in this study correspond to the sphere-behavior of other cell types in literature and suggest a similar and characteristic tissue-like manner [18].

CLSM analysis and the proof of actin and FAC inside all fully developed spheres proved the intimate cell-cell contacts as shown in the literature for spheres of other cell types such as osteoblasts and mesenchymal stem cells [18,19,39]. Moreover, the dense network of stained actin and FAC inside the spheres (Figure 2) confirms the tissue-like cell interaction shown in the images of the scanning electron microscopic investigations.

Of special interest is the ability of single cells to grow out of the sphere after its attachment on different substrates. This is an elementary requirement for the use of spheres as testing method for biomaterials or as a possible 3-D tissue equivalent cell formation for tissue engineering and tissue regeneration similar to this study.

Actin and FAC staining of the outgrowing cells represent a dense actin skeleton, FAC and equally round shaped cell nuclei, which are signs for vital cell metabolism. The radial and consistent outgrowth of the cells at the contact area between the spheres and substrate indicates the same developmental stage of the pulp cells, which form a fully developed sphere. Furthermore, the outgrown sphere cells emphasize the tissue-like character and cell vitality of the pulp spheres. The feature of cells growing out of tissue explants is well known and used as a cell extraction strategy for primary cell cultures.

Further evidence for the tissue-like character of the pulp spheres used for the first time in this study is the ability of several spheres to merge and build new and tissue-like cell agglomerations. As shown in the results, these new constructs build up a common surface very similar to the outer layer of an individual sphere that formed a new cell structure which had the ability to grow around and cover biomaterials [18].

In this study, the cell spheres were cultured on bovine and in human root canals to analyze their reaction towards a physiological environment.

Directly after the spheres attached onto the dentin surface, single cells started growing out of the cell agglomerate, spread out flat and aligned themselves on the dentin structure (Figures 5 and 6). Following the surface orientated migration (Figure 6a) cell processes grew into dentinal tubules followed by a complete coverage of the internal tubule walls (Figure 6b). This progress, from the outgrown cells over the surface orientated migration up to the complete dentinal coating of the root canal surface and the walls of the dentinal tubules, clarifies the ability of the pulp cells after being pre-differentiated into tissue-like spheres to interact with their original physiological environment. It seems to be a considerable advantage that the cells grew out of the spheres in a very high number. Shao et al. also report about a multilayered cell construct that was formed in a two-dimensional cell culture system on dentin slabs after four days of cultivation [12]. The application of spheroidal, micromass cultures as performed in this study, showed a direct and multilayered outgrowth of the cells from the pulp spheres on the root canal wall as well as into the dentinal tubules (Figures 5 and 6).

The results of the *in vivo-*study of Kodonas et al. showed an odontoblast-like cell construct with an organic matrix formation on the root canal wall surface of porcine teeth after ten weeks [27]. The prepared teeth had been treated with porcine dental pulp stem cells. In the present study, a phenomenon regarding the multilayered cell accumulation on the root dentin surfaces occurred, which had not been described before in studies dealing with pulp tissue regeneration. After 28 days, a three-dimensional tubules-formation was detected within the multilayered cell accumulation simulating the root dentin surface underneath (Figures 5 and 6). It seems that these cell structures represent a higher level of cell organization as previously shown in other studies where multiple cell layers were described. The newly three-dimensional emulated dentin-like tissue presents tubule structures and the beginning of physiological dental hard tissue formation. Not only multilayered cell constructs that have been described in literature before, but also newly build three-dimensional structures with cell-cell contacts and extracellular matrix formation over its entire volume were detected in the present study.

The findings of other studies have shown that dentin structures, and the release of different bioactive agents embedded in dentin, such as transforming growth factor-β can induce and provide an odontogenic differentiation of cells despite the use of disinfectants during endodontic root canal treatment [27]. In this study, the dentinal substrate seems to stimulate the cell growth out of the pre-differentiated spheres to form cell-cell contacts, to develop extracellular matrix and to create dentin tissue-like three-dimensional structures. The use of the pre-differentiated three-dimensional cultured cells [24] may enhance the stimulation of mantle dentin or tertiary dentin formation respectively, and might have a substantial impact on pulp capping and partial pulp removal in endodontics. This kind of tissue regeneration is also promoted by the calcium ion release of different pulp capping materials [40].

Interestingly, the use of the spheres in contact with the human dentin initiated a detectable biomineral formation. 28 days after seeding the spheres into the human root canals and the tissue like fusion of the spheres mineral formation was proven by SEM investigation (SE- and BSE-detection) and EDX without any additional treatment with differentiation factors. The fact that the mineral particles detected under the sphere surface covered by cell layers (Figure 4) leads to the conclusion that the mineral is formed inside the spheres and is transported out of the spheres to its surface. Lammers et al. have shown mineral formation starting inside the spheres by osteogenically stimulated stem cell spheres [39].

The cell caused mineralization is a clear sign of a fast and highly advanced differentiation of the “pulp cells” into a mineral/dentin producing cell type. To the best knowledge of the authors, dentin like tissue formation and comparable mineral formation on spheres surfaces as presented in this study have not been shown until now. This applies for the use of human dental pulp cell spheres in contact with dentin. The potential clinical application of spheres appears very promising, but the impact of disinfective irrigants on the spheres or of residual bacteria needs to be investigated.

## Conclusion

The current study represents a significant step in the characterization and use of spheres and their high potential for dental tissue regeneration and scaffold free pulp tissue engineering due to their proven ability to emulate dentin like tissue and to initiate biomineralization.

## Competing interests

The authors declare that they have no competing interests.

## Authors’ contributions

JN performed cell culture, light microscopic analysis, SEM- and EDX-investigation and carried out the conception of this study and the manuscript. MTW and GW prepared the human and bovine root specimen, provided and cultivated the DPC and drafted and wrote parts of the manuscript. CH, GL and HPW contributed to the conception, design and coordination of the study and manuscript as well as the interpretation of the data. The study was performed under supervision of HPW. All authors read and approved the final manuscript.
